# Application effect of short-term traffic flow prediction method based on CNNBLSTM algorithm

**DOI:** 10.1371/journal.pone.0327460

**Published:** 2025-07-07

**Authors:** Guozhu Sui, Meixia Song, Ke Bian, Mingzhen Zhang, Xiaogang Zhang, Yiru Wang

**Affiliations:** School of Traffic and Electrical Engineering, Dalian University of Science and Technology, Dalian, China; University of Queensland - Saint Lucia Campus: The University of Queensland, AUSTRALIA

## Abstract

Reduced forecast efficiency and accuracy are the result of traditional traffic flow prediction algorithms’ inability to adequately capture the spatiotemporal characteristics and dynamic changes of traffic flow. To address this problem, this study proposes a short-term traffic flow prediction method based on an improved convolutional neural network and a bidirectional long short-term memory algorithm. The method firstly identifies, repairs and decomposes the abnormal traffic flow data by smoothing the estimation threshold and adaptive noise integration empirical modal decomposition method to improve the data quality and stability. The suggested model is then supplemented with the enhanced Adam and Lookahead algorithms in an effort to increase the model’s prediction accuracy and rate of convergence. The outcomes indicated that the method showed faster convergence and lower loss values during both training and validation. The training loss decreased from 0.0250 to 0.0021, and the validation loss decreased from 0.0010 to 0.0008. Compared with the traditional convolutional neural network with bidirectional long short-term memory algorithm, the training loss decreased by 42.86% The suggested algorithm outperformed the current advanced algorithms in terms of prediction precision, with an average absolute percentage error of 0.233 and a root mean square error of 23.87. The findings display that the study’s suggested algorithm can effectively and precisely forecast the short-term traffic flow, which serves as a solid foundation for planning and traffic management decisions.

## 1. Introduction

Due to the acceleration of urbanization and the increase in traffic volume, traffic congestion has become a major barrier to urban growth in the modern period. Because severe traffic congestion increases the likelihood of traffic accidents, accurate traffic flow prediction (TFP) is crucial for traffic management and planning [1]. However, traditional TFP methods, although they can provide prediction results to a certain extent, are difficult to effectively capture the spatial- temporal (ST) characteristics and dynamic changes of TF. This makes the prediction efficiency low and the accuracy of prediction results poor [2]. Therefore, recently, there has been a lot of interest in looking into an accurate and effective TFP technique. The quick advancement of deep learning (DL) technology in recent years has given rise to a fresh approach to TFP [3]. In the realm of temporal dynamics, the combination of convolutional neural networks (CNNs) with bidirectional long short-term memory (Bi-LSTM) has attracted a lot of interest and applications. To better interpret sequence data, this technique combines CNN’s spatial feature extraction capabilities with LSTM’s time-dependent modeling capabilities [4].

The majority of academics and professionals have studied short-term TFP (STTFP) techniques due to the quick development of machine learning (ML) and deep learning (DL). Z. Wu et al. proposed a DL TFP model based on improved meta-heuristic optimization. The model utilized an improved augmented extreme gray wolf optimizer and combined with Bi-LSTM for CNN. The outcomes revealed that the model outperformed existing methods in terms of goodness of fit, mean absolute percentage error (MAPE) [5]. To address the issue of traffic congestion in cities during peak hours, A. Navarro-Espinoza et al. suggested a TFP approach based on ML and DL algorithms. According to the findings, every model put forth in the research performed better in terms of predictions [6]. W. Shu et al. proposed an enhanced gate recurrent unit neural network for a STTFP model. The model employed an improved bidirectional positive and negative feedback gate recurrent unit for STTFP, and the optimizer was improved using a corrective adaptive model. The model performed better in TFP, according to the data [7]. Q. Chen et al. suggested an upgraded wavelet neural network-based STTFP model to take into consideration the nonlinear and time-varying characteristics of traffic. The wavelet neural network prediction technique’s sluggish convergence and local optimum issues were resolved by the model using the particle swarm optimization approach. The outcomes revealed that this algorithm produced more accurate and consistent prediction results [8]. X. Zhu et al. propose a TFP model based on multi-viewpoint ST convolution. The input coding layer taught the model how to represent sequence data, and it successfully integrated time and location data to enhance its prediction capabilities. According to the findings, the model’s MAPE increased by roughly 1.2% [9]. An successful STTFP approach was proposed by T. D. Toan et al. To increase the prediction precision (PP), the model was trained using an enhanced closest neighbor technique and support vector machine. The outcomes revealed that the K-nearest neighbor (KNN) method accelerated the training speed without affecting the prediction performance [10].

In recent years, DL has been widely applied in various fields, such as image recognition, speech recognition, natural language processing, medical diagnosis, and financial risk prediction, due to its powerful ability to model complex nonlinear relationships [11]. In the field of transportation, traditional traffic flow prediction methods, such as time series analysis, regression models, and statistical based models, often struggle to capture complex features such as spatiotemporal characteristics, dynamic changes, and nonlinear characteristics of traffic flow, resulting in reduced prediction accuracy and efficiency [12]. Deep learning techniques can process large-scale, high-dimensional data and extract meaningful spatiotemporal patterns. Therefore, in the field of transportation, deep learning technology is particularly valued [13]. CNN and Bi-LSTM, as DL techniques, are widely used in TFP because of their ability to effectively capture the time series dependence and nonlinear features of TF data (TFD) [14]. In the field of transportation, CNN has been applied to traffic flow prediction by processing traffic data as images or spatial sequences. For example, researchers use CNN to extract spatial features from traffic data collected by sensors distributed along the road network [15]. Then, these spatial features are used as inputs for other models for further prediction. Bi LSTM is an extension of LSTM that processes data in both forward and reverse directions [16]. This enables the model to capture past and future dependencies in the data, which is particularly useful for traffic flow prediction as the current traffic state is influenced by past and future traffic conditions. Z. Cheng et al. presented the STTFP technique, which uses a vector autoregression (VAR) model to assess the intrinsic correlation between traffic data. Moreover, a CNN-LSTM hybrid neural network model was utilized to predict multi-feature speed at a spatial location. The results indicated that the model predictionprecision was closely related to the spatial correlation of TF [17]. S. Lu et al. suggested an innovative short-term TF (STTF) combination prediction method to address the complexity and stochasticity of TF. The findings showed that the suggested approach produced accurate prediction results [18]. C. Ma et al. suggested a TFP method based on TF time series analysis with Bi-LSTM algorithm. The technique used the enhanced Bi-LSTM network for learning and training. According to the findings, the approach performed better in terms of accuracy and stability than the conventional approach [19]. To solve the issue of classic TFP models failing to take spatial correlation into account, W. Zhuang et al. suggested a STTFP model based on KNN and Bi-LSTM networks. The station data was spatially filtered by the model using the KNN method before being sent into the Bi-LSTM network for prediction. The outcomes demonstrated a 22% improvement in the model’s performance [20]. A multi-step prediction model (PM) for STTF based on CNN and Bi-LSTM networks was proposed by W. Zhuang et al. for quickly increasing traffic data. The model took the spatial features of TFD as inputs to the Bi-LSTM network as a way to efficiently extract the time series features of traffic. The results indicated that the average absolute error of the model was reduced by 30.4% [21]. Z. Li et al. proposed a new TFP method which combines Bi-LSTM network with an attention mechanism in an effort to improve the representation of key TF information. The outcomes revealed that the root mean square error (RMSE) and MAPE index of the method were reduced by 1.6%−4.7% and 0.6%−22.8%, respectively, and had a high PP [22]. The summary of the methods and their advantages and disadvantages in current research is shown in Table 1.

**Table 1:** Summary of the methods in current research.

Model	Advantages	Disadvantages	References
Machine learning and deep learning for traffic control	Enhanced fitting accuracy, lower MAPE and MAE	Complexity	Z. Wu et al. [5]
Machine learning and deep learning for traffic control	Adaptive traffic control	May require more computational resources	A. Navarro-Espinoza et al. [6]
Improved GRU with bidirectional feedback	Efficient short-term prediction	Limited to short-term	W. Shu et al. [7]
Improved wavelet neural network	More precise and stable predictions	Computationally intensive	Q. Chen et al. [8]
Multi-view spatiotemporal convolution	Improved prediction accuracy by 1.2%	Complexity	X. Zhu et al. [9]
Support vector machine with k-nearest neighbors	Faster training speed	May not capture complex patterns well	T. D. Toan et al. [10]
CNN-LSTM for spatial-temporal prediction	High prediction accuracy related to spatial correlation	High computational cost	Z. Cheng et al. [17]
Rolling regression and LSTM	Good prediction effect	Limited to linear features	S. Lu et al. [18]
Time series analysis with Bi-LSTM	Better accuracy and stability	Requires large datasets	C. Ma et al. [19]
K-nearest neighbors and Bi-LSTM	Improved performance by 22%	High computational cost	W. Zhuang et al. [20]
CNN and Bi-LSTM for multi-step prediction	Reduced MAE by 30.4%	Complexity	W. Zhuang et al. [21]
Bi-LSTM with attention mechanism	Higher prediction accuracy	Requires more data	Z. Li et al. [22]

Overall, although some achievements have been made in the field of traffic flow prediction, there are still research gaps. first. Most existing research mainly focuses on optimizing a single traffic flow prediction model, such as improving the Bi LSTM model or combining it with other optimization algorithms to improve prediction accuracy. However, there are few studies that delve into multi model fusion strategies to fully utilize the advantages of different models to further improve prediction accuracy and stability. Secondly, most existing research is based on historical traffic data for prediction, and insufficient consideration is given to the impact of real-time traffic events on traffic flow. These unexpected events may significantly alter the spatiotemporal characteristics of traffic flow, leading to a decrease in the prediction accuracy of existing models. In addition, with the continuous development of intelligent transportation systems, the scale and complexity of traffic data continue to increase. However, current research still faces challenges in processing large-scale, multi-source heterogeneous traffic data, lacking efficient data fusion and processing methods [23].

In view of this, the study proposes a STTFP method based on CNN and Bi-LSTM (CNNBLSTM) algorithm, which aims to improve the TFP effect and precision. The innovations of this research are (1) The empirical modal decomposition (EEMDAN) method with smoothed estimation threshold and adaptive noise integration is used to improve the data quality and stability of TF. (2) To increase the system’s efficiency, the Radam optimization algorithm dynamically modifies the learning rate (LR) based on the CNNBLSTM model’s dynamics during the training phase. (3) The model’s convergence speed (CS) is expedited, its generalization ability (GA) and robust performance are enhanced, and the learning process is made more stable by using the Lookahead optimization approach.

The contribution of this study is the proposal of a short-term traffic flow prediction method that combines improved CNN and Bi LSTM. This method can efficiently and accurately predict the trend of traffic flow changes, providing real-time and reliable decision support tools for traffic management departments. By accurately predicting traffic flow, it is possible to optimize traffic signal control, alleviate traffic congestion, and reduce the risk of traffic accidents, thereby improving the operational efficiency and management level of urban transportation systems.

## 2. Methods and materials

This section first proposes a TFD identification and decomposition method based on smoothing estimation threshold and EEMDAN, which aims to repair and decompose abnormal TFD. Then a STTFP model based on improved CNNBLSTM algorithm is constructed to improve the PP of the model.

### 2.1. TFD identification and decomposition method based on smoothing estimation threshold and eemdan

TFD is crucial for traffic state identification, traffic management and control. With the development of traffic information collection systems, massive TFD are continuously emerging [24]. However, due to the nonlinear, cyclic, mobile, and random characteristics of TFD, there are complex ST characteristics between its collected data, and the data also have the problems of incompleteness, error, and poor quality. This will affect the accurate prediction of TF [8]. Therefore, the study suggests a TFD identification and decomposition approach with smoothing estimation threshold and EEMDAN to guarantee the input of normal and stable TFD and enhance the PP of STTF. The method firstly uses the smoothing estimation threshold method to identify the abnormal TFD in depth, and repairs the abnormal TFD by using historical data, neighboring data and ST correlation. Then, the repaired TFD are processed and denoised by adaptive noise integration empirical modal decomposition method. Moreover, it is decomposed into features of different time scales as a way to solve the nonlinearity and nonsmoothness problem of this data.

Abnormal TFD is mainly caused by the intermittency of the record, which will make gaps in the time series, thus affecting the continuity of the data, and outlier data will affect the prediction results of TF. The study first uses the independent threshold method to initially identify the abnormal TFD, and the more obvious abnormal data are excluded [25]. The erroneous TFD is then deeply identified by smoothing the estimation threshold. The study defines the sequence data of TF as y(t) . The median of y(m−2), y(m−1), y(m), y(m+1), y(m+2) is defined as $\dot{y}(m)$, and y(m−2) is eliminated. Then the median of y(m−1), y(m), y(m+1), y(m+2), y(m+3) is defined as $\dot{y}(m+1)$, and the values are taken gradually. A new sequence of TF $\dot{y}(t)$ is proposed to be processed by this smoothing. For the new sequence $\dot{y}(t)$, the study defines the median of $\dot{y}(m-1)$, $\dot{y}(m)$, $\dot{y}(m+1)$ as $\ddot{y}(m)$, and eliminates $\dot{y}(m-1)$. Then the median of $\dot{y}(m)$, $\dot{y}(m+1)$, $\dot{y}(m+2)$ is defined as $\dot{y}(m+1)$, which is taken step by step to establish the new sequence of TF $\ddot{y}(t)$ with the secondary smoothing treatment. Then the computation of the new sequence of TF with the tertiary smoothing treatment is shown in Equation (1).


$$\dddot{y}(m)=\frac{1}{4}\ddot{y}(m-1)+\frac{1}{4}\ddot{y}(m+1)-\frac{1}{2}\ddot{y}(m)$$ (1)

In Equation (1), $\dddot{y}(t)$ is the new sequence of TF after three smoothing processes. Then the study fits the interval of TFD sampling with RMSE by exponential function to establish the minimum and maximum threshold model. The expression is shown in Equation (2).


$$\left\{ \begin{array}{c} Y_{\min}=Y-g(T)\\ Y_{\max}=Y+g(T) \end{array}\right.$$ (2)

In Equation (2), $Y_{\min}$ denotes the minimum critical threshold. $Y_{\max}$ denotes the maximum critical threshold. Y denotes the estimated value of the smoothing process. g(T) denotes the function that adjusts the thresholds to adapt to the changes in the data. When the actual measured TFD is within the range of the threshold value, it indicates that the data is reasonable, otherwise the data is abnormal. For the processing of actual measured traffic flow data, the study first thoroughly cleans the collected actual traffic flow data to remove any damaged or incomplete records that may affect the accuracy of the analysis. For outliers, the study used the quartile range method for filtering, and missing values were filled in using the time domain averaging method. In order to capture the temporal dynamics and spatial dependencies in traffic flow data, additional features have been engineered. These features include time of day, number of days in the week, and holiday indicators, which are known to affect traffic patterns. The processed data is divided into a training set, a validation set, and a testing set. This partitioning allows the model to be trained on one subset of data, adjusted on another subset of data, and ultimately evaluated on a separate dataset to ensure the model’s generalization ability. After the anomalous data is deeply recognized by the above method, it needs to be further repaired. The study uses the historical data method to count the TFD of the previous moment and the adjacent moment. Its calculation is shown in Equation (3) [26].


$$E_{x_{u}}(f)=\eta x_{u}(f)+(1-\eta )E_{x_{u}}(f-7)$$ (3)

In Equation (3), $E_{x_{u}}(f)$ denotes the historical data value of TF on day f under sampling intervals u. $x_{u}(f)$ denotes the actual TFD value. η denotes the smoothing coefficient. $E_{x_{u}}(f-7)$ denotes the corresponding actual data of the last week. The study corrects the TFD with contiguous data anomalies by forecasting the TF at the time of prediction. The calculation is shown in Equation (4).


$$X(t)=\frac{x(t-n)+\ldots +x(t-1)+x(t+1)+\ldots +x(t+n)}{2n}$$ (4)

In Equation (4), X(t) represents the value of abnormal TFD after repair. n represents the quantity of neighboring data. For the repair of abnormal TFD of detector data, the study is carried out using ST correlation. The expression is shown in Equation (5).


$$\left\{ \begin{array}{c} X_{u}(k,a,b)=\gamma_{0}+\gamma_{1}x_{u}(a)+\gamma_{2}x_{u}(b)\\ X_{u}(k)=median\left[X_{u}(k,a,b)\right] \end{array}\right.$$ (5)

**Fig 1:**
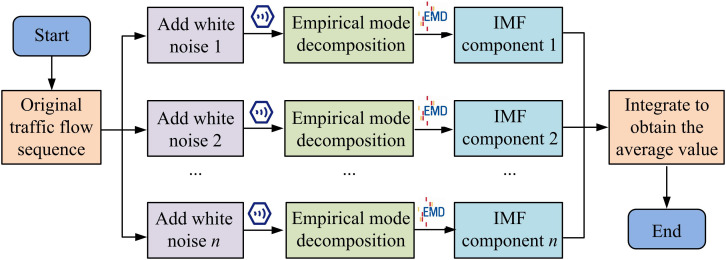
Schematic diagram of ensemble empirical mode decomposition.

In Equation (5), $X_{u}(k,a,b)$ denotes the restored value of position k under TFD detectors a and b. $X_{u}(a)$ and $X_{u}(b)$ denote the measured data of location and position. $X_{u}(k)$ is the predicted value (PV) of the detector in the missing case. $\gamma_{0}$, $\gamma_{1}$, and $\gamma_{2}$ denote the regression equation coefficients. The EEMDAN approach is examined to address the noise residual issue of TFD after correcting the inaccurate and missing data. The intrinsic mode function (IMF) obtained from the decomposition is filtered to minimize the decomposition error and to eliminate local skew signals [27]. In this method, the white noise (WN) $\varepsilon_{0}V_{1}\omega^{i}(t)$ of the TFD is firstly added to its sequence x(t), and the data is decomposed by the empirical mode decomposition algorithm. The decomposition schematic of the method is shown in Fig 1.

After passing the decomposition, the obtained IMF components (IMFC) need to be optimized and averaged to obtain the first stage IMFCs of the improved EEMDAN algorithm. Equation (6) provides the expression for this process.


$$\left\{ \begin{array}{c} {\bar{C}}_{1}(t)=\frac{1}{N}\sum_{i=1}^{N}C_{1}^{i}(t)\\ R_{1}(t)=x(t)-{\bar{C}}_{1}(t) \end{array}\right.$$ (6)

In Equation (6), ${\bar{C}}_{1}(t)$ is the first-stage IMFC of the EEMDAN algorithm. $C_{1}^{i}(t)$ denotes the first stage IMFC obtained by the empirical modal decomposition algorithm. N is the sequence length of the TF. $R_{1}(t)$ denotes the residual signal after the first decomposition. x(t) is the original TF sequence at time t. In WN $\varepsilon_{0}V_{1}\omega^{i}(t)$, $\varepsilon_{0}$ denotes the intensity coefficient of the first WN in the TF sequence. $V_{1}$ is the variance of the first WN. $\omega^{i}(t)$ is the WN signal. Then the residual signal continues to be introduced into the WN and decomposed again to obtain the IMFC of the second order and the j th residual signal, as calculated in Equation (7).


$$\left\{ \begin{array}{c} {\bar{C}}_{2}(t)=\frac{1}{N}\sum_{i=1}^{N}V_{1}\left[R_{1}(t)+\varepsilon_{1}V_{1}\omega^{i}(t)\right]\\ R_{j}(t)=R_{j-1}(t-{\bar{C}}_{j}(t)) \end{array}\right.$$ (7)

In Equation (7), ${\bar{C}}_{2}(t)$ is the second order IMFC obtained by the EEMDAN algorithm. $R_{j}(t)$ denotes the j th residual signal. $\varepsilon_{1}$ denotes the intensity coefficient of the second WN. ${\bar{C}}_{j}(t)$ denotes the IMFC of the j th order. Then the calculation of the j+1 th order IMFC obtained by the EEMDAN algorithm and the final residual signal at the end of the decomposition is shown in Equation (8).


$$\left\{ \begin{array}{c} {\bar{C}}_{j+1}(t)=\frac{1}{N}\sum_{i=1}^{N}V_{1}\left[R_{1}(t)+\varepsilon_{j}V_{j}\omega^{i}(t)\right]\\ R(t)=x(t)-\sum_{j=1}^{J}{\bar{C}}_{j}(t) \end{array}\right.$$ (8)

**Fig 2:**
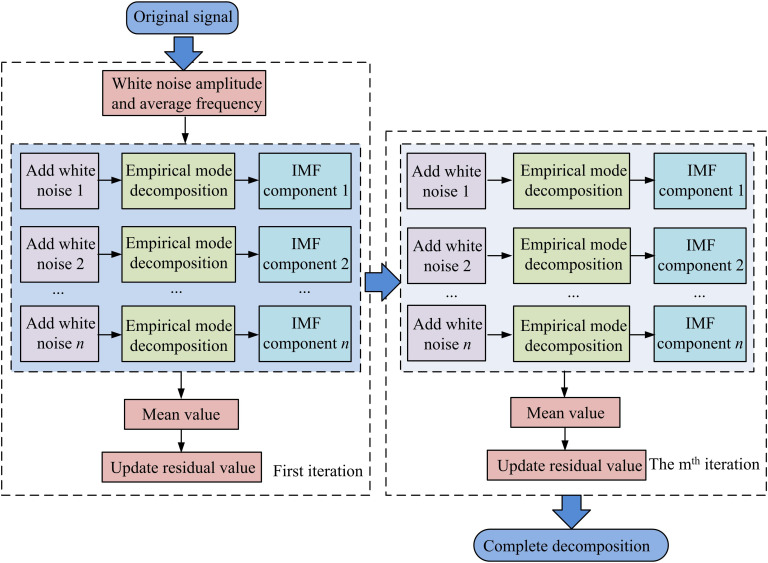
Schematic diagram of ensemble empirical mode decomposition with adaptive noise.

In Equation (8), ${\bar{C}}_{j+1}(t)$ is the j+1 th order IMFC result. $\varepsilon_{0}$ denotes the intensity coefficient of the j th WN in the TF sequence. $V_{j}$ denotes the variance of the j th WN. R(t) denotes the final residual signal at the end of decomposition. ${\bar{C}}_{j}(t)$ denotes the j th order IMFC result. The decomposition flow of this EEMDAN method is schematically shown in Fig 2.

### 2.2. STTFP model construction based on improved cnnblstm algorithm

After identifying and decomposing the anomalous TFD by smoothing estimation threshold and EEMDAN method, the study needs to further process the data. With the rapid development of DL, the CNNBLSTM algorithm is widely used for its ability to effectively extract ST features from TFD [28]. However, the existing CNNBLSTM algorithm has high computational complexity, low LR and slow CS. Moreover, a more intricate network topology will have an impact on the model’s GA [29]. Therefore, the study improves the existing algorithm and constructs a new STTFP model. To increase the PM’s computational efficiency and robustness, the method combines the Lookahead optimization algorithm with the modified Adam optimization algorithm to improve the CNNBLSTM algorithm.

The presence of temporal, spatial, and periodic variations in TF will impact the model’s prediction accuracy. As a result, the study must be normalized prior to model training, which is determined using Equation (9).


$$S^{\prime }=\frac{S-T_{fd\min}}{T_{fd\max}-T_{fd\min}}$$ (9)

**Fig 3:**
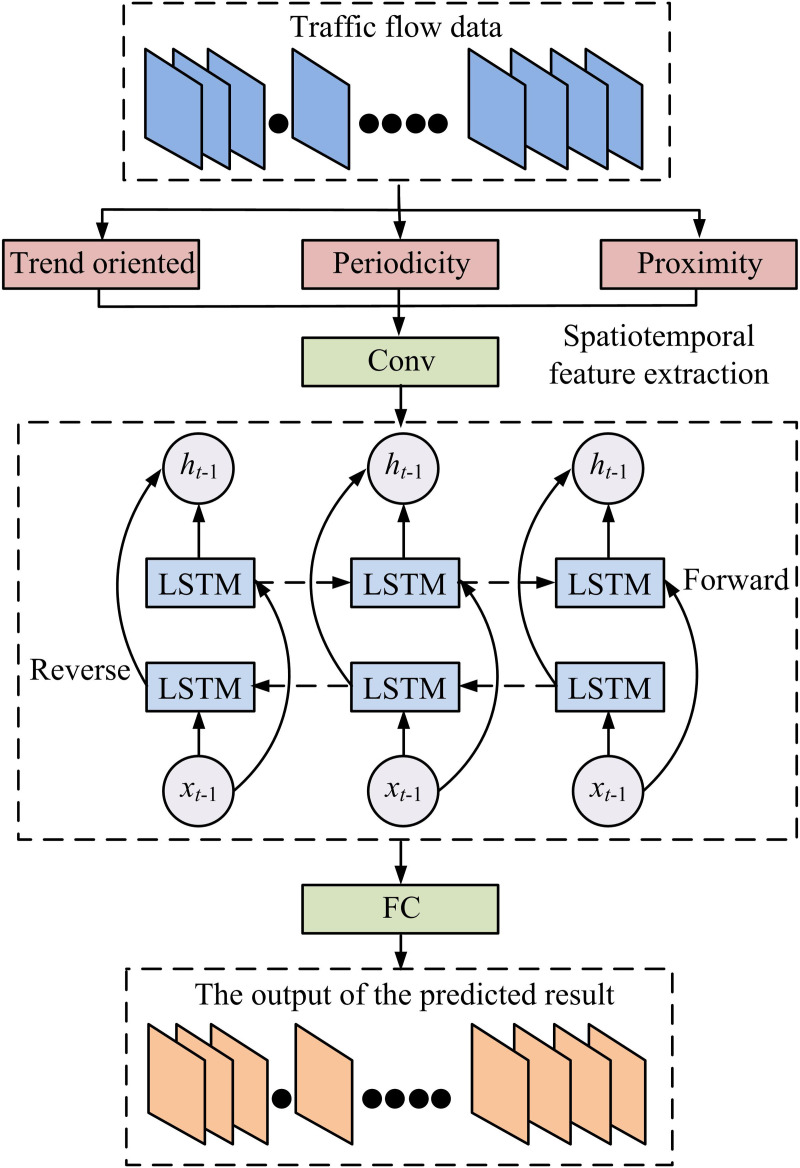
Flowchart of CNNBLSTM algorithm.

In Equation (9), $S^{\prime }$ is the normalized TFD. S is the original TFD. $T_{fd\max}$ and $T_{fd\min}$ denote the highest and lowest values of TF samples. For the training of the model, the study first processes the TF time series of the normalization operation to construct a kind of feature matrix that integrates ST correlations [30]. Then, the spatial features of the TFD are extracted using CNN. The method automatically extracts the spatial features of the TF sequence through the construction of convolutional and pooling layers. The feature vector of the TF sequence is then obtained by feeding the feature matrix into the multilayer CNN. The feature vector is then fed into the Bi-LSTM in an attempt to capture the temporal information. Lastly, the model is trained using batchsizw and epoch parameters to get the model’s prediction results. Fig 3 schematically depicts the model’s flow.

In the TP of the PM, when the training sample data is insufficient, the parameters of the PM will be affected by the first few samples, reducing the convergence of the PM. To resolve the above problems, the study uses the improved Adam optimization algorithm to effectively modify the method of adaptive LR [31]. The method first performs an initialization operation on the moving average $v_{t}$ of the momentum $m_{t}$ of the first-order moment estimation (1st-ME) and the gradient squared of the second-order moment estimation (2rd-ME). Among them, $m_{t}=0$, $v_{t}=0$, and the time step (TS) t=1. Then, in each iteration, the gradient $g_{t}$ is computed and the 1st-ME and the 2rd-ME are updated. The expression is shown in Equation (10).


$$\left\{ \begin{array}{c} g_{t}=\Delta_{\delta }f_{t}(\delta_{t-1})\\ m_{t}=\beta_{1}\cdot m_{t-1}+(1-\beta_{1})\cdot g_{t}\\ v_{t}=\beta_{2}\cdot v_{t-1}+(1-\beta_{2})\cdot g_{t}^{2} \end{array}\right.$$ (10)

In Equation (10), $g_{t}$ denotes the gradient at step t. $\delta_{0}$ is the initial parameters of the input. f(δ) denotes the optimization function. $\beta_{1}$ and $\beta_{2}$ denote the decay rate of the 1st-ME and 2rd-ME, respectively. After updating the 1st-ME and 2rd-ME, their bias corrections need to be further computed. Their calculation is shown in Equation (11).


$$\left\{ \begin{array}{c} {m^{\prime }}_{t}=\frac{m_{t}}{1-\beta_{1}^{t}}\\ {v^{\prime }}_{t}=\frac{v_{t}}{1-\beta_{2}^{t}} \end{array}\right.$$ (11)

In Equation (11), $m_{t}^{\prime }$ and $v_{t}^{\prime }$ denote the 1st-MEs and 2rd-MEs after bias correction is performed, respectively. Then, the study dynamically adjusts the LR and updates the parameters based on the data from model training. The calculation is shown in Equation (12).


$$\left\{ \begin{array}{c} \rho \;\infty =\frac{2}{1-\beta_{2}}-1\\ \rho_{t}=\rho \;\infty -\frac{2t\beta_{2}^{t}}{1-\beta_{2}^{t}}\\ R_{t}=\sqrt{\frac{\left(\rho_{t}-4\right)\left(\rho_{t}-2\right)\rho \;\infty }{\left(\rho \;\infty -4\right)\left(\rho \;\infty -2\right)\rho_{t}}}\\ \delta_{t}=\delta_{t}-1-\eta_{t}\cdot \frac{{m^{\prime }}_{t}}{\sqrt{{v^{\prime }}_{t}}+\sigma } \end{array}\right.$$ (12)

**Fig 4:**
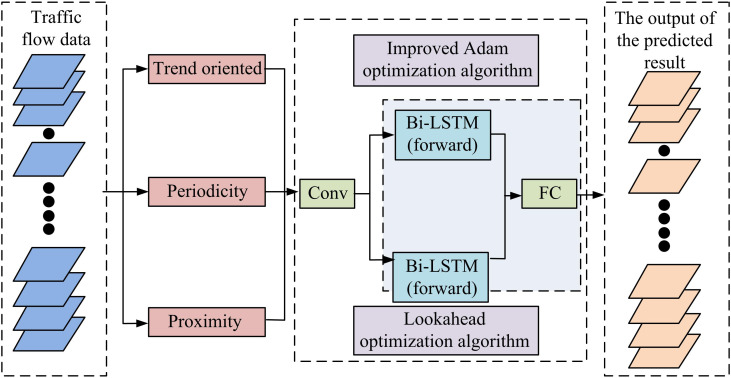
Diagram of the improved CNNBLSTM model.

In Equation (12), ρ∞ denotes the decay rate function for 2rd-ME. $\rho_{t}$ denotes the dynamic LR adjustment factor at the TS. $R_{t}$ denotes the dynamic LR adjustment factor. δ denotes the updated model parameters. $\eta_{t}$ denotes the LR. σ denotes numerical stability constant. To address the issue of the model’s sluggish convergence, the study incorporates the Lookahead optimization method into the PM in an effort to increase the model’s rate of convergence and prediction accuracy [32]. Fig 4 illustrates the schematic of the enhanced CNNBLSTM model.

Lookahead optimization algorithm improves the stability and CS of model training by maintaining the fast and slow weights. The method starts by setting the initial parameters as $\varsigma_{0}$ and the objective function as L. The synchronization period and the slow weight step size are k and α, respectively. The optimizer is A. Equation (13) provides the expression that the optimizer uses to adjust the fast and slow weights [33].


$$\left\{ \begin{array}{c} \lambda_{j,i+1}=\lambda_{j,i}+A(L,\lambda_{j,i-1},d)\;i=1,2,\ldots ,k\\ \phi_{j+1}=\phi_{j}+\alpha (\lambda_{j,k}-\phi_{j}) \end{array}\right.$$ (13)

In Equation (13), $\lambda_{j,i+1}$ and $\phi_{j+1}$ denote the updated fast weights and slow weights, respectively. i and j are the quantity of updates and training times of the optimizer, respectively. After updating the weights, further judgment on the error and training times is required. If the condition is satisfied then it is the final weights, otherwise the update needs to be repeated. The study uses a quadratic loss function (LF) to approximate the objective function in order to derive the optimal slow weighting step size so as to effectively reduce the loss, which is calculated as displayed in Equation (14).


$$\left\{ \begin{array}{c} L(x)=\frac{1}{2}x^{T}Bx-b^{T}x\\ \alpha^{\prime }=\mathrm{\backslash arg}\min_{\alpha }L(\lambda_{t,0}+\alpha (\lambda_{t,k}-\lambda_{t,0})) \end{array}\right.$$ (14)

In Equation (14), L(x) denotes the quadratic LF. B denotes the symmetric positive definite matrix. b denotes the gradient of the LF. $\alpha^{\prime }$ denotes the optimal slow weight step. $\lambda_{t,0}$ is the initial value of fast weights at TS t. $\lambda_{t,k}$ is the fast weights at TS t and after the k th fast weight update. Finally, the study utilizes the curvature quadratic approximation for the optimal step size as shown in Equation (15) [34].


$$\alpha^{\prime \prime }=clip\left(\frac{\left(\lambda_{t,0}-{\left({\dot{A}}^{-1}\nabla L\left(\lambda_{t,k}\right)\right)}^{T}\dot{A}\left(\lambda_{t,0}-\lambda_{t,k}\right)\right)}{{\left(\lambda_{t,0}-\lambda_{t,k}\right)}^{T}\dot{A}\left(\lambda_{t,0}-\lambda_{t,k}\right)}\right)$$ (15)

**Fig 5:**
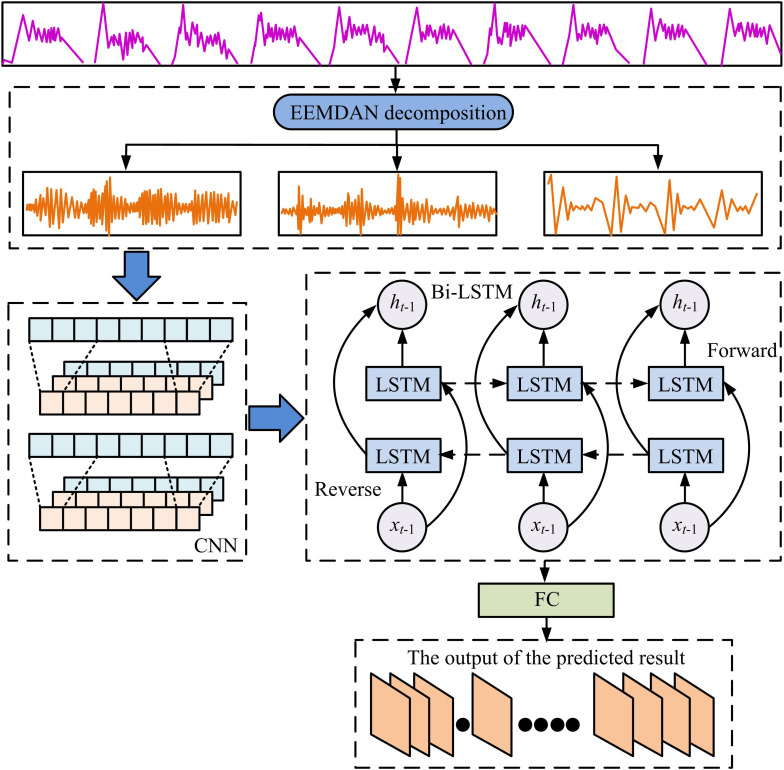
Schematic diagram of STTFP model.

In Equation (15), $\alpha^{\prime \prime }$ denotes the optimal step size. $\dot{A}$ denotes the approximate Hessian matrix. $\nabla L(\lambda_{t,k})$ denotes the gradient of the LF. After adding the above optimization algorithm to the PM, it is further trained. Fig 5 displays the completed STTFP model’s flow diagram.

## 3. Results

The performance and efficacy of the repair and distinct techniques for TFD are initially examined in this section. The enhanced CNNBLSTM algorithm’s performance is then confirmed, and the usefulness of using this PM is examined.

### 3.1. Identification, restoration, and analysis of decomposition results of TFD

The study conducts experiments using the performance measurement system (PeMS) dataset, which provides TF and speed data for the California freeway system. TFD for a particular week in the dataset is intercepted for the experiment.

**Fig 6:**
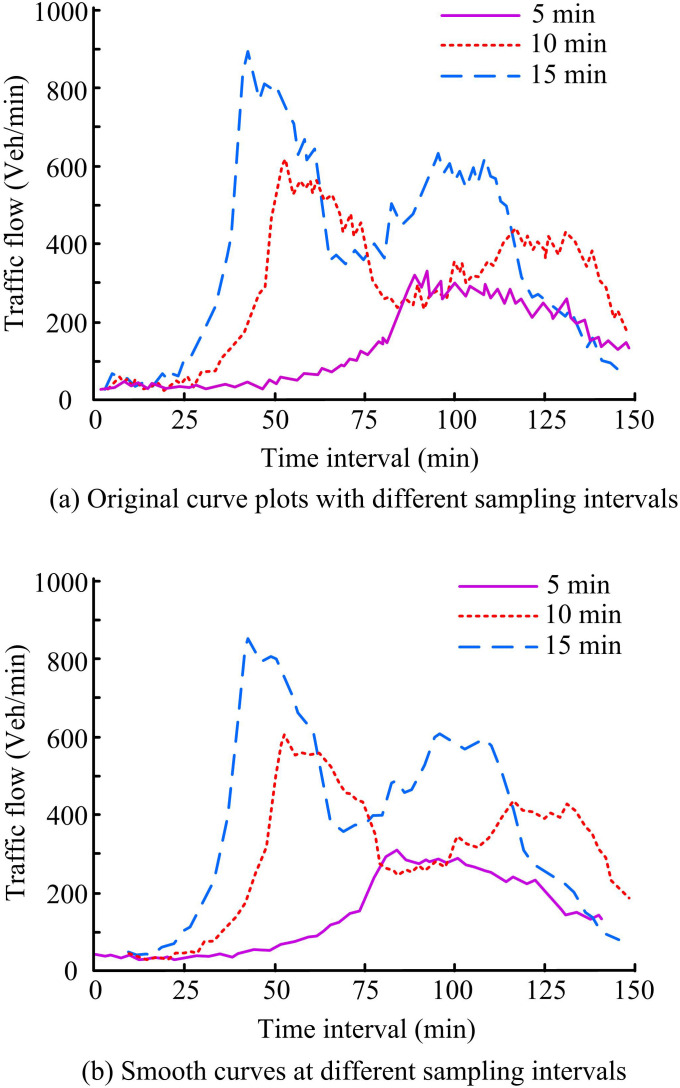
Comparison of raw curves with different sampling intervals and smoothed curves.

A comparison of the original and smoothed TF curves at various time points is the first step in the investigation. Fig 6 displays the findings. In Fig 6(a), it is demonstrated that when the sampling intervals are 5 min, the peak value of TF is 307.28 Vel/min. When the sampling intervals are 10 min and 15 min, the peak values of TF are 604.15 Vel/min and 890.84 Vel/min, respectively. The outcomes reveal that the fluctuation of TF is more obvious under shorter sampling intervals and some noise is generated during the sampling process. In Fig 6(b), it is shown that when the sampling intervals are 5 min and 10 min, the TF after the smoothing treatment is 298.72Vel/min and 590.24 Vel/min, respectively. When the sampling intervals are 15 min, its TF is 867.58 Vel/min. The results show that, in comparison to the data before smoothing, the suggested smoothing technique can successfully enhance data quality and lessen the impact of outliers. Moreover, the data can effectively provide high-quality input data for the subsequent DL model.

**Fig 7:**
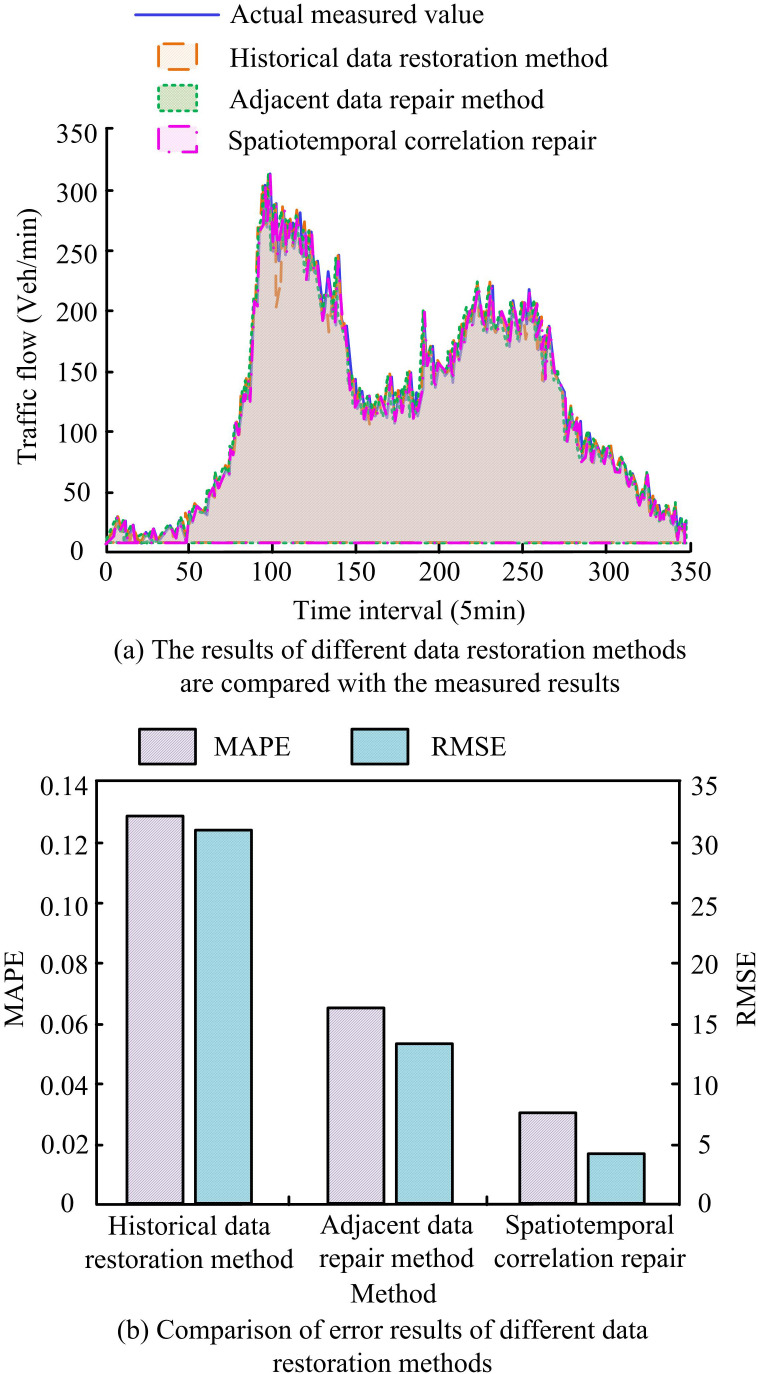
Comparison of repair results and error results of different data repair methods.

The study examines the effects of various data restoration techniques and evaluates restoration result errors, such as RMSE and MAPE. Fig 7 displays the findings. In Fig 7(a), the actual measured TF is 221.17 Vel/min when the time is 100 min. The TF of the historical data restoration method and the adjacent data restoration method are 180.25 Vel/min and 192.87 Vel/min, respectively. Compared with the actual measured TF, they are reduced by 18.50% and 12.80%, respectively. The TF of the ST correlation repair method is 217.62 Vel/min. In Fig 7(b), the MAPE and RMSE values of the historical data repair method are 0.128 and 31.126, respectively. The MAPE values of the neighboring data repair method and the ST correlation repair method are 0.063 and 0.032. The RMSE values are 13.782 and 4.917. Comparing the ST correlation repair approach to the historical data repair and surrounding data repair methods, the MAPE values are lowered by 75.00% and 49.21%. There is an 84.20% and 64.32% reduction in the RMSE values. The findings indicate that the ST correlation repair method has the smallest error with the measured values, which proves that the method has a high repair precision.

**Fig 8:**
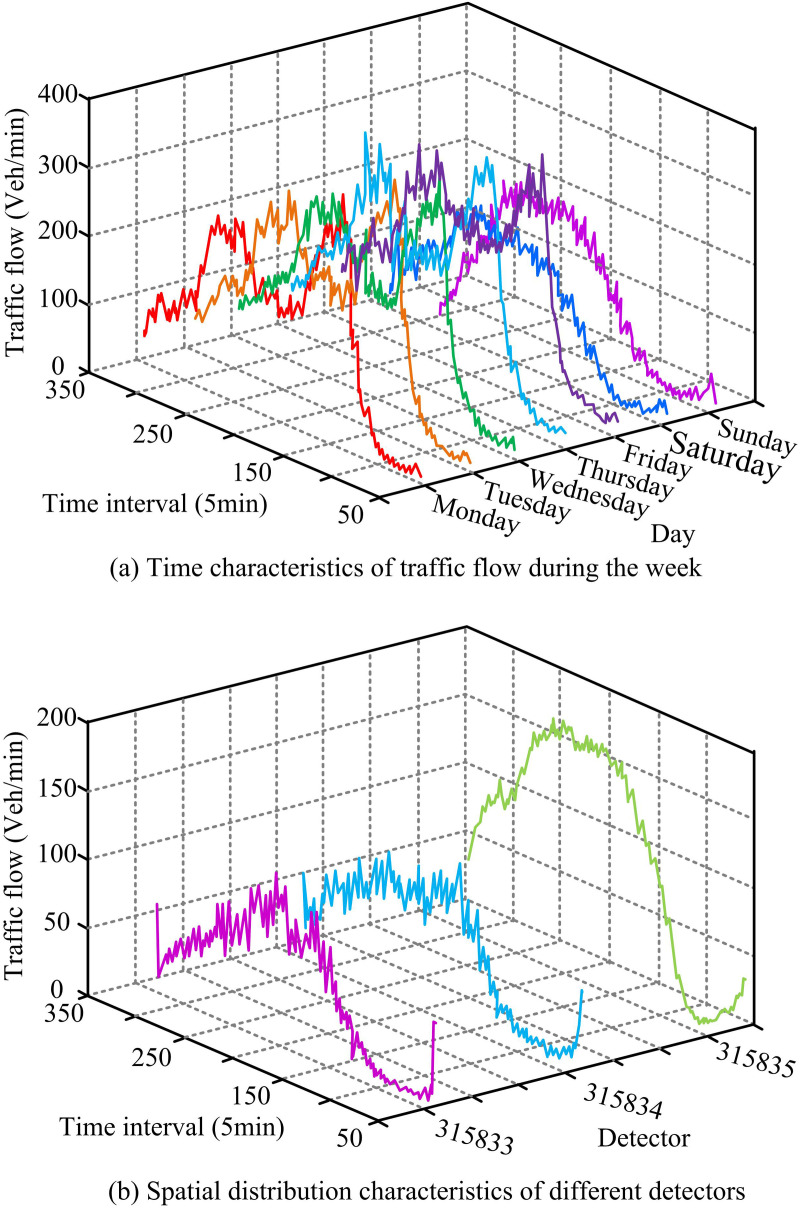
Analysis of ST characteristics of TF.

To better anticipate future TFs, the study examined the ST features of the TFD to identify cyclical variations in the data. Figure displays the findings. In Fig 8(a), during working days, the peak TF phenomenon occurs at 7:00–9:00 AM and 17:00–19:00 AM, and the TF is about 200–300 Vel/min and 250–300 Vel/min, respectively. On non-working days, the morning peak is significantly offset by the evening peak, with traffic volumes of about 150–200 Vel/min. The results show that the peak flow on working days is significantly higher than that on weekends, showing strong regularity and periodicity. The weekend flow is relatively stable with less fluctuation and can provide the main basis for the prediction of STTF. In Fig 8(b), both detectors 315833 and 315834 have a TF of about 50–100 Vel/min in the peak period. Detector 315835 has a TF of about 150–200 Vel/min in the peak period. Traffic planning can benefit from the data, which show that the distribution of TF varies depending on the site.

**Fig 9:**
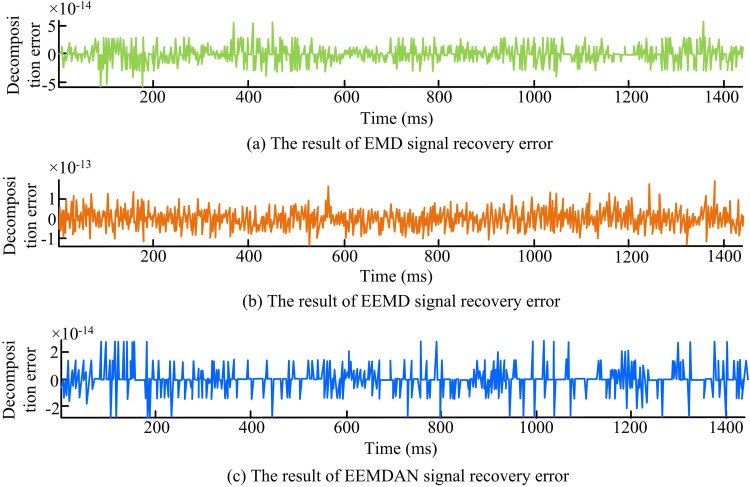
Comparison of reconstruction error results of three decomposition method.

The study compares the reconstruction error of the EEMDAN method with that of the EMD and EEMD methods in an attempt to validate the decomposition performance of the method. Fig 9 displays the findings. In Fig 9(a), it is shown that the reconstruction error of the EMD method fluctuates widely, with error values ranging from −5 × 10^-14^ to 5 × 10^-14^. This Figure shows that the EMD method is unstable in the error value in the time series, and there is an obvious high-frequency oscillation phenomenon, which is not able to effectively remove the noise in the data. In Fig 9(b), the fluctuation of the EEMD method is improved compared with the EMD method, and the fluctuation range is narrowed between −1 × 10^-13^ and 1 × 10^-13^. The results illustrate that the method solves part of the endpoint effect problem by adding random noise, but the inability distribution is still not smooth enough. In Fig 9(c), the reconstruction error of the proposed EEMDAN method is significantly reduced between −2 × 10^-14^ and 2 × 10^-14^. Furthermore, the error values in the time series are smoother, and the high-frequency oscillations are basically eliminated. The findings illustrate that by adaptively modifying the noise level, the suggested approach may successfully prevent noise interference and accumulated errors.

### 3.2. Performance validation of improved CNNBLSTM algorithm and analysis of prediction results

To verify the efficacy of the proposed PM, the study uses the K-fold cross-validation approach to establish the model’s parameters. In the model’s TP, 10% of the sample data is used as the validation set, 20% as the test set, and 70% as the training set. Table 2 displays the model parameter settings as well as the experimental setup.

**Table 2:** Setting of experimental environment and model parameters.

Experimental environment		Model parameter setting	
Hardware environment		Improved Adma LR	0.001
System	Windows 10	LR of Lookahead	0.5
Storage	16GB/1T	Filters	64
Processor	Intel(R)core(TM)i5-10400@2.90 GHz	Batch size	32
Software environment		Kernel size	6
Framework	Keras	Pool size	4
Python	3.7	LSTM output size	70
Keras	2.2.5	Strides	2
Tensorflow	1.14	Dropout rate	0.5

**Fig 10:**
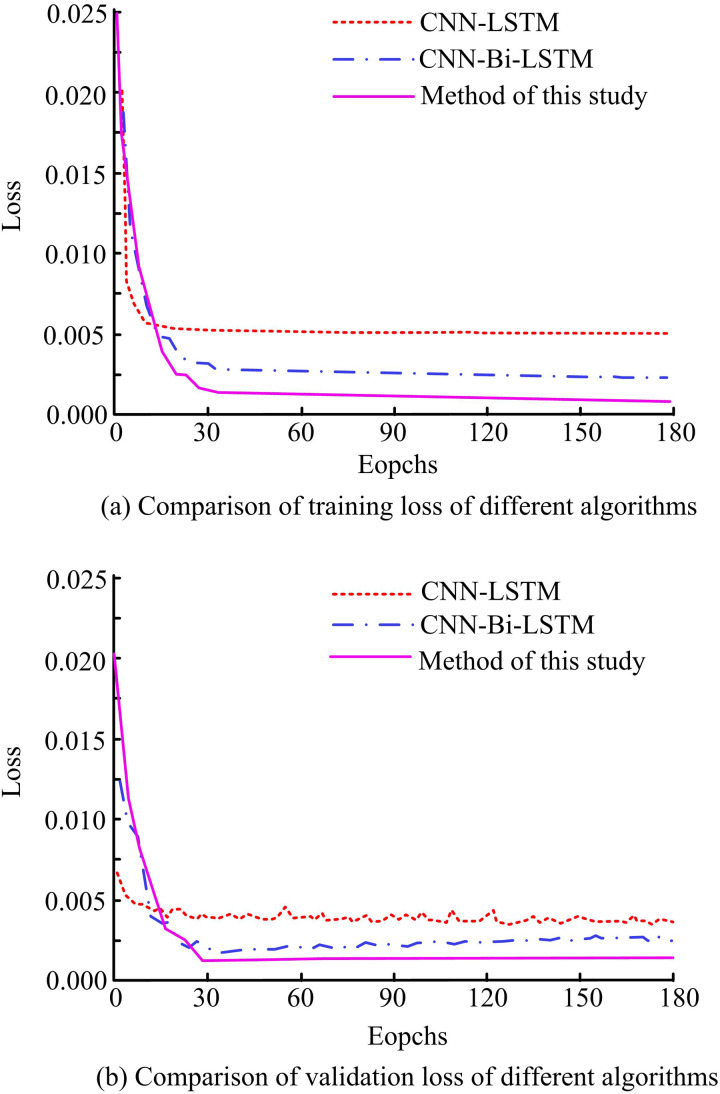
Comparison of TL and VL of different algorithms.

To confirm the efficacy of the suggested approach, the study first examines the training and validation losses (VLs) of several algorithms. Fig 10 displays the findings. In Fig 10(a), when the iteration is 30, the training loss (TL) of CNN-LSTM algorithm decreases from 0.0202 to 0.0063, that of CNNBLSTM algorithm decreases from 0.0179 to 0.0028, and that of the study proposed algorithm decreases from 0.0250 to 0.0021. When the iteration is increased to 90, the TL of the three algorithms is 0.0058, 0.0028, and 0.0016, respectively. The TL of the suggested algorithms is decreased by 75.72% and 42.86%, respectively, in comparison to the CNN-LSTM algorithm and the CNNBLSTM method. In Fig 10(b), in the validation set, when the iteration is 30, the losses of the CNN-LSTM algorithm and the CNNBLSTM algorithm are 0.0042 and 0.0023, respectively. The loss of the study-proposed algorithm is 0.0010. When the iteration reaches 90, the losses of the three algorithms are 0.0039, 0.0022, and 0.0008, respectively. The outcomes reveal that studying the proposed algorithm shows faster convergence during both training and validation, and the loss of this algorithm is better than the other algorithms.

**Fig 11:**
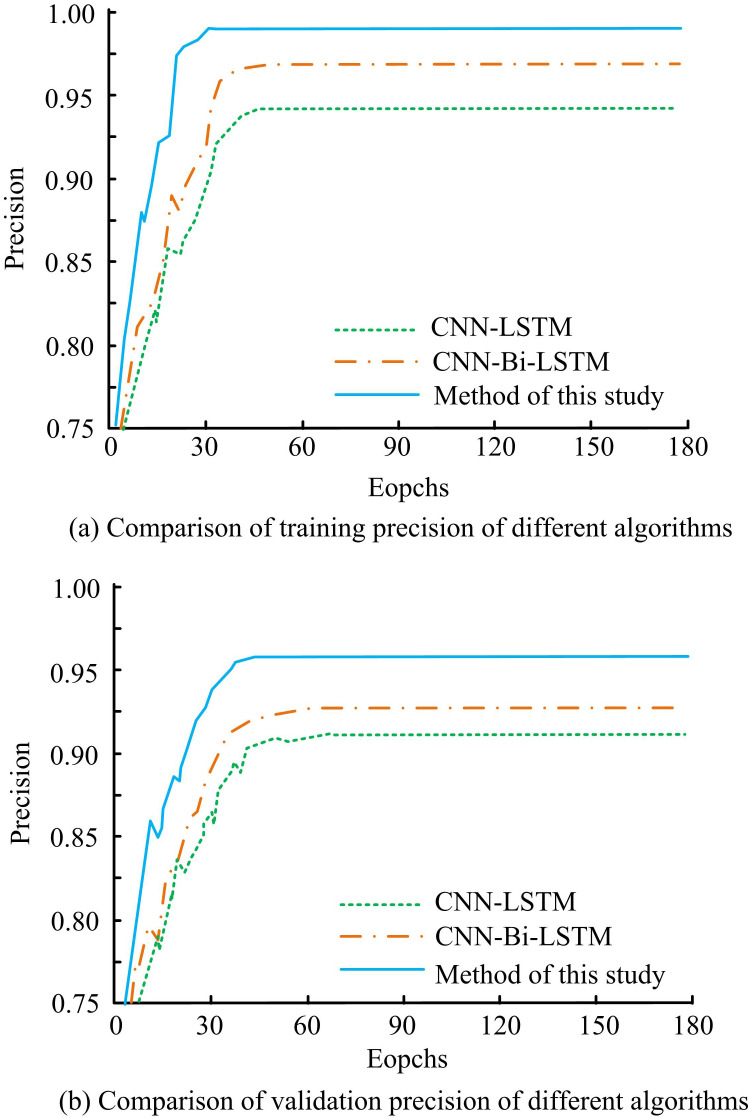
Comparison of training precision and validation precision of different algorithms.

The study examines the training and validation precisions prior to and following the algorithm improvement in an attempt to confirm the viability of the suggested approach. Fig 11 displays the findings. In Fig 11(a), during the training of the model, when the iteration is 30, the precision of the CNN-LSTM algorithm, CNNBLSTM algorithm, and the study of the proposed algorithm are 0.872, 0.921, and 0.979, respectively. When the iteration is increased to 90, the precision of the CNN-LSTM algorithm and CNNBLSTM algorithm are 0.937 and 0.968, respectively. In comparison, the precision of the RLI algorithm is 0.982, which is an improvement of 4.58% and 1.43% over the other two algorithms’ precisions, respectively. In Fig 11(b), during the validation of the algorithms, when the iteration is 30, the precision of the CNN-LSTM algorithm and CNNBLSTM algorithm are 0.850 and 0.876, respectively. The precision of the algorithms proposed by the study is 0.934. When the iteration is increased to 90, the three algorithms have a precision of 0.905, 0.925, and 0.954. The precision of the studied proposed algorithms is improved by 5.14% and 3.04% compared to the CNN-LSTM and CNNBLSTM algorithms, respectively. The outcomes reveal that the proposed algorithm is studied to have superior precision in both training and validation processes.

**Fig 12:**
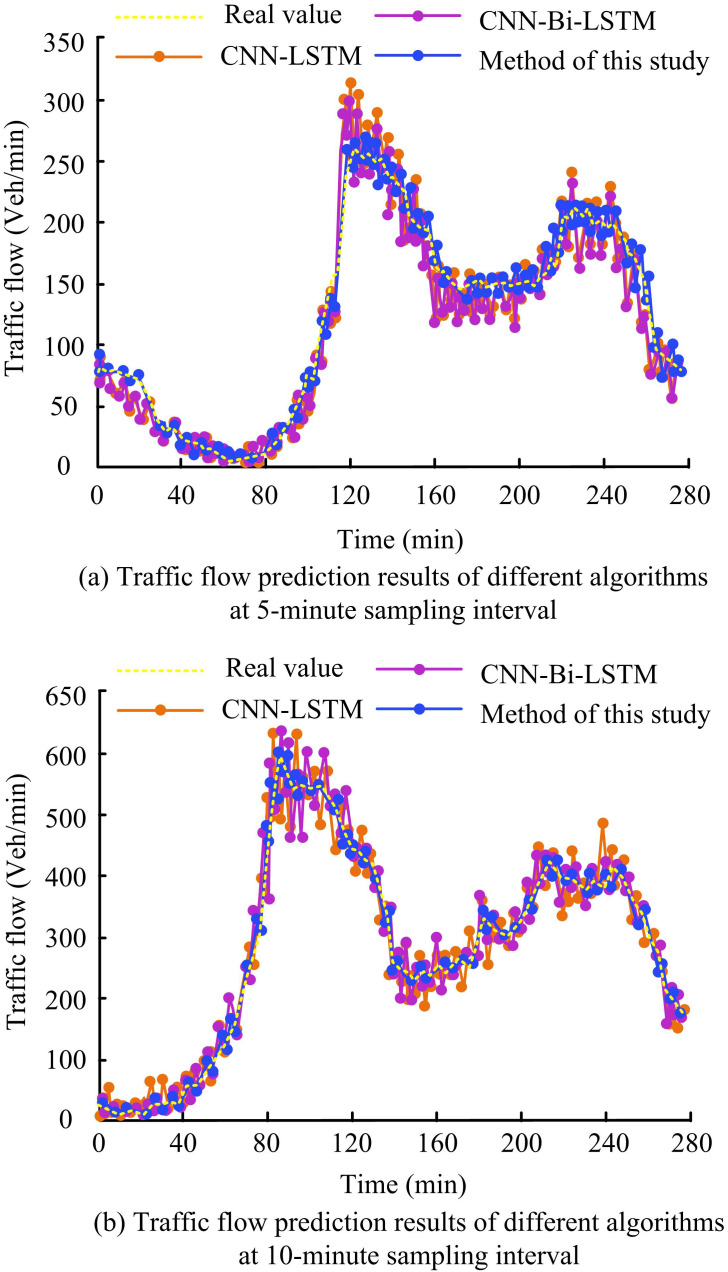
TFP results of different algorithms at different sampling intervals.

To examine the efficacy of the suggested algorithm for STTFP, the study also compares the TFP outcomes of various algorithms at various time points. Fig 12 illustrates the findings. In Fig 12(a), when the sampling intervals are 5 min, the actual TF for a time of 120 min is about 260 Veh/min. The TF predicted by CNN-LSTM and CNNBLSTM is about 315 Veh/min and 300 Veh/min, respectively. In comparison to it, the TF predicted by the proposed method is studied to be about 265 Veh/min, which is 5 Veh/min different from the actual TF. It is found that the PVs of the proposed model are well fitted to the actual values with high PP. In Fig 12(b), the actual TF at time 90 min for sampling intervals of 10 min is about 598 Veh/min. The TF predicted by CNN-LSTM, CNNBLSTM, and the study of the proposed model is about 618 Veh/min, 620 Veh/min, and 602 Veh/min, respectively. The outcomes reveal that the proposed model has higher TFP precisions at different sampling intervals.

**Fig 13:**
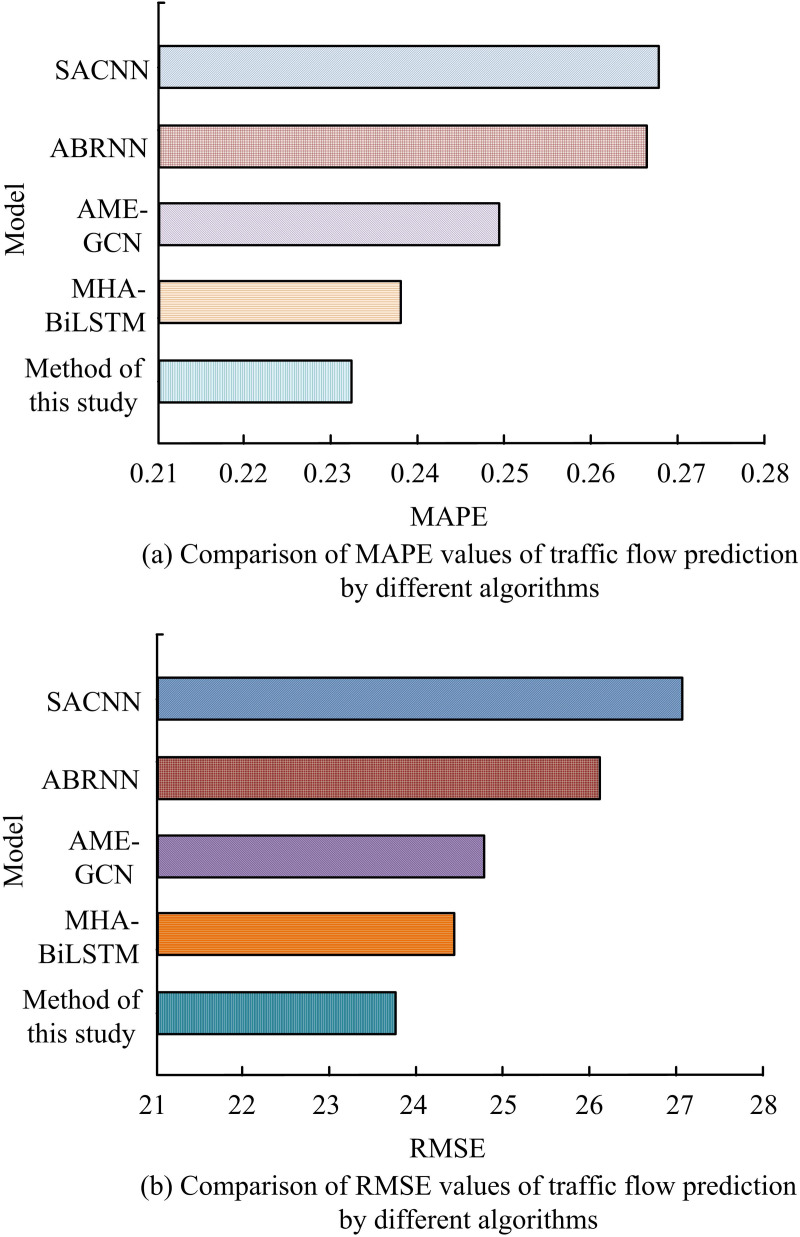
Comparison of MAPE value and RMSE value of traffic flow prediction by different algorithms.

In order to verify the predictive performance of the proposed algorithm for traffic flow, the study analyzed the prediction error of the algorithm compared with existing advanced algorithms, including Self Attention Convolutional Neural Network (SACNN), Attention Based Recurrent Neural Network (ABRNN), Attention Mechanism Enhanced Graph Convolutional Network (AME-GCN), and Multi Head Attention Bidirectional Long Short Term Memory (MHA BiLSTM). The results are shown in Fig 13. As shown in Fig 13(a), the MAPE values of SACNN and ABRNN algorithms are 0.268 and 0.266, respectively, while the MAPE values of AME-GCN, MHA BiLSTM, and the proposed algorithm are 0.249, 0.238, and 0.233, respectively. Compared with the other four algorithms, the MAPE of the proposed algorithm decreased by 13.06%, 12.41%, 6.43%, and 2.10%, respectively. As shown in Fig 13(b), the RMSE values of SACNN, ABRNN, AME-GCN, and MHA BiLSTM algorithms are 27.32, 26.28, 24.82, and 24.57, respectively. Compared with it, the RMSE value of the algorithm proposed by the research institute is 23.87, which is 12.63%, 9.17%, 3.83%, and 2.85% lower than the other four algorithms, respectively. The results indicate that the predictive performance of the proposed algorithm is superior to other advanced algorithms.

**Fig 14:**
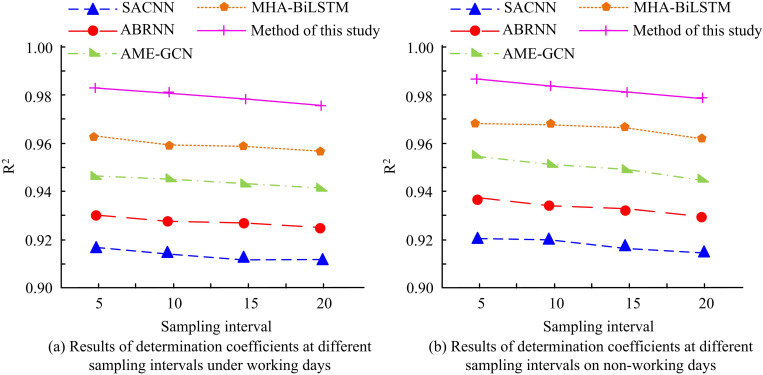
Results of determination coefficients at different sampling intervals on working days and non-working days.

In order to further explore the superiority of the proposed algorithm, the determination coefficient R2 of working days and non working days on different sampling interval datasets was analyzed and compared with other popular algorithms. The results are shown in Fig 14. As shown in Fig 14(a), on weekdays, the determination coefficients of SACNN, ABRNN, and AME-GCN algorithms are 0.918, 0.930, and 0.947 at a sampling interval of 5 minutes, and 0.914, 0.929, and 0.945 at a sampling interval of 15 minutes, respectively. The determination coefficients of MHA BiLSTM and the algorithm proposed by the research institute are 0.963 and 0.982 at a sampling interval of 5 minutes, and 0.961 and 0.978 at a sampling interval of 15 minutes, respectively. In Fig 14(b), it was found that on non working days, the determination coefficients of SACNN, ABRNN, AME-GCN, MHA BiLSTM, and the proposed algorithm were 0.920, 0.937, 0.955, 0.969, and 0.988 at a sampling interval of 5 minutes, and 0.918, 0.933, 0.951, 0.968, and 0.982 at a sampling interval of 15 minutes, respectively. The results indicate that the proposed algorithm has a high coefficient of determination and the best fitting ability in short-term traffic flow prediction on both working and non working days.

To verify the progressiveness of the proposed method, the research compares the performance of different prediction methods, including CNN-LSTM, Temporal Graph Convolutional Network (T-GCN), Transformer based Traffic Flow Forecasting (TB-TFF), Spatial Temporal Graph Neural Network (STGNN) and Adaptive Graph Convolutional Recurrent Network (AGCEN). The results are shown in Table 3. As shown in Table 3, the proposed method performs the best in all indicators. MAPE angle CNN-LSTM decreased by 13.1%, and RMSE decreased by 12.6%. In terms of coefficient of determination, the proposed method has an R^2^ of 0.982 at 5-minute intervals and 0.978 at 15 minute intervals, both of which are higher than other methods, demonstrating better fitting and prediction accuracy.

**Table 3:** Performance comparison of different prediction methods.

Methods	MAPE	RMSE	R^2^(5 min)	R^2^ (15 min)
CNN-LSTM	0.268	27.32	0.918	0.914
T-GCN	0.251	25.63	0.972	0.965
TB-TFF	0.247	24.92	0.975	0.969
STGNN	0.239	24.15	0.980	0.967
AGCEN	0.241	24.08	0.979	0.914
OURS	0.233	23.87	0.982	0.978

To further validate the proposed method, additional experiments were conducted using real-world traffic flow data under different conditions, including weekday peak, weekday off peak, weekend peak, weekend off peak, and holiday. The results are summarized in Table 4 below. Table 4 shows that the model proposed in this study performs well in different traffic scenarios. During peak hours on weekdays, MAPE is 0.228, RMSE is 22.45, and R^2^ values are close to 0.981 and 0.976. The performance is better during non peak hours, with MAPE dropping to 0.215, RMSE at 20.87, and R^2^ values as high as 0.984 and 0.980. The prediction accuracy for weekends and holidays slightly decreased, but the R^2^ values remained above 0.973 and 0.969, verifying the robustness and adaptability of the model.

**Table 4:** Performance of the proposed model in real-world scenarios.

Scenario	MAPE	RMSE	R^2^(5 min)	R^2^ (15 min)
Weekday Peak	0.228	22.45	0.981	0.976
Weekday Off-Peak	0.215	20.87	0.984	0.980
Weekend Peak	0.231	23.12	0.978	0.974
Weekend Off-Peak	0.208	19.76	0.986	0.982
Holiday	0.242	24.53	0.973	0.969

## 4. Discussion and conclusion

To enhance the CS and PP of the PM, the study suggested a short-time TFP technique based on the enhanced CNNBLSTM algorithm. The study’s findings revealed that the smoothed TF dropped by 9.56 Vel/min, from 307.28 Vel/min to 298.72 Vel/min, when the sampling intervals were set at 5 min. It was shown that the method was effective in smoothing the data fluctuations and providing more stable data inputs for the subsequent model. The suggested PM’s TL dropped from 0.0250 to 0.0021 during the TP. The training precision was 0.982, which was 1.34% higher than the traditional CNNBLSTM algorithm. In the TFP results of different sampling intervals, the difference between its predicted TF and the actual value was only 5Veh/min. It indicated that the TF predicted by this method fitted well with the actual TF. Compared with SACNN, ABRNN, AME-GCN, and MHA BiLSTM algorithms, the MAPE values of the proposed algorithm decreased by 13.06%, 12.41%, 6.43%, and 2.10%, respectively, and the RMSE values decreased by 12.63%, 9.17%, 3.83%, and 2.85%, respectively, demonstrating the superiority of the algorithm.

In recent years, deep learning methods based on attention mechanisms have made significant progress in the field of short-term traffic flow prediction. These methods significantly improve prediction accuracy by enhancing the model’s ability to focus on key spatiotemporal features. However, the method proposed in this study still has unique advantages in terms of convergence speed, prediction accuracy, and robustness compared to these latest algorithms.

H. The prediction model proposed by Y. Kan et al. has achieved significant improvements in accuracy, but its convergence speed is relatively slow. In contrast, the method proposed by the research institute combines Adam optimization algorithm with Lookachead optimization algorithm, and the accuracy of the model is as high as 0.954 with a loss of 0.0008. Its accuracy and convergence performance are significantly better than H Y. The model proposed by Kan et al. Z. Li et al. combined Bi LSTM with attention mechanism, which performs well in indicators such as MAPE and RMSE. However, it requires high data preprocessing and lacks robustness in handling non-stationary traffic flow data. This study preprocessed the data using the EEMDAN method, and the error values were between −2 × 10^-14^ and 2 × 10^-14^. The reconstruction error was significantly lower than Z The method proposed by Li et al.

In summary, the suggested approach can greatly increase the model’s PP and perform better in STTFP, which can more precisely forecast the TF trend. This study’s limitations include its very small data set and its failure to account for variables like weather. Therefore, it is necessary to further explore the effectiveness of this method in larger datasets and complex traffic scenarios in future studies.

## Supporting information

S1 Data Minimal Data set. (DOC)
