# Supplementary material for: Application effect of short-term traffic flow prediction method based on CNNBLSTM algorithm
Source: PLoS One. 2025 Jul 7;20(7):e0327460. doi: 10.1371/journal.pone.0327460 (PMC12233303; doi:10.1371/journal.pone.0327460)
Supplement: S1 Data — (DOC) [file pone.0327460.s001.doc]

**The data in Figure 6**

| Time interval (min) | Traffic flow of original curve plots (Vel/min) | | | Traffic flow of smooth curves (Vel/min) | | |
| --- | --- | --- | --- | --- | --- | --- |
|  | 5 min | 10 min | 15 min | 5 min | 10 min | 15 min |
| 1 | 20.26 | 20.04 | 20.19 | 42.17 | 0.00 | 0.00 |
| 25 | 21.57 | 69.56 | 98.67 | 36.57 | 52.45 | 103.58 |
| 50 | 41.25 | 604.15 | 796.23 | 62.19 | 590.24 | 805.25 |
| 75 | 123.58 | 439.26 | 372.59 | 200.04 | 412.27 | 385.48 |
| 100 | 298.57 | 361.57 | 582.46 | 286.56 | 325.74 | 598.58 |
| 125 | 258.66 | 394.12 | 275.45 | 216.39 | 398.56 | 266.58 |
| 150 | 146.87 | 182.16 | 72.14 | 139.86 | 200.02 | 90.25 |

**The data in Figure 7**

| Time interval (5min) | Actual measured value (Vel/min) | Historical data restoration method (Vel/min) | Adjacent data repair method (Vel/min) | Spatiotemporal correlation repair (Vel/min) |
| --- | --- | --- | --- | --- |
| 0 | 0.00 | 0.00 | 0.00 | 0.00 |
| 50 | 16.27 | 21.28 | 8.79 | 24.20 |
| 100 | 221.17 | 180.25 | 192.87 | 217.62 |
| 150 | 123.69 | 126.47 | 125.46 | 142.56 |
| 200 | 158.75 | 157.56 | 158.37 | 159.66 |
| 250 | 182.46 | 202.47 | 173.89 | 170.28 |
| 300 | 77.28 | 78.59 | 72.59 | 75.47 |
| 350 | 19.87 | 20.36 | 22.15 | 9.86 |
| MAPE | / | 0.128 | 0.063 | 0.032 |
| RMSE | / | 31.126 | 13.782 | 4.917 |

**The data in Figure 8(a)**

| Time interval (5min) | Monday (Vel/min) | Tuesday (Vel/min) | Wednesday (Vel/min) | Thursday (Vel/min) | Friday (Vel/min) | Saturday (Vel/min) | Sunday (Vel/min) |
| --- | --- | --- | --- | --- | --- | --- | --- |
| 50 | 12.82 | 11.47 | 12.56 | 25.48 | 23.62 | 15.66 | 16.87 |
| 100 | 58.92 | 92.45 | 59.96 | 58.27 | 67.72 | 56.65 | 62.57 |
| 150 | 184.35 | 195.46 | 175.36 | 177.47 | 169.96 | 168.72 | 176.83 |
| 200 | 216.58 | 258.96 | 255.49 | 268.57 | 245.63 | 159.46 | 186.25 |
| 250 | 124.68 | 139.65 | 148.59 | 160.25 | 170.58 | 125.60 | 108.96 |
| 300 | 269.57 | 266.65 | 277.26 | 278.54 | 298.65 | 192.84 | 196.75 |
| 350 | 82.67 | 68.58 | 77.44 | 66.25 | 55.63 | 39.24 | 48.28 |

**The data in Figure 8(b)**

| Time interval (5min) | Detector 315833 (Vel/min) | Detector 315834 (Vel/min) | Detector 315835 (Vel/min) |
| --- | --- | --- | --- |
| 50 | 50.24 | 48.57 | 32.41 |
| 100 | 36.86 | 39.56 | 90.15 |
| 150 | 96.57 | 75.86 | 125.47 |
| 200 | 102.45 | 82.48 | 158.96 |
| 250 | 84.24 | 44.69 | 169.42 |
| 300 | 45.12 | 52.55 | 98.59 |
| 350 | 52.15 | 49.56 | 40.14 |

**The data in Figure 9**

| Time (ms) | Decomposition error | | |
| --- | --- | --- | --- |
|  | EMD | EEMD | EEMDAN |
| 0 | 0.00 | 0.62×10^-13^ | -0.87×10^-14^ |
| 200 | -2.81×10^-14^ | 0.08×10^-13^ | -1.36×10^-14^ |
| 400 | 0.00 | -0.74×10^-13^ | -1.35×10^-14^ |
| 600 | -0.98×10^-14^ | -0.25×10^-13^ | 0.56×10^-14^ |
| 800 | -0.12×10^-14^ | 0.10×10^-13^ | -2.00×10^-14^ |
| 1000 | -2.37×10^-14^ | -0.41×10^-13^ | 0.00 |
| 1200 | 0.00 | -0.53×10^-13^ | -0.63×10^-14^ |
| 1400 | -1.05×10^-14^ | -0.42×10^-13^ | -2.00×10^-14^ |

**The data in Figure 10**

| Epochs | Training loss | | | Training loss | | |
| --- | --- | --- | --- | --- | --- | --- |
|  | CNN-LSTM | CNN-Bi-LSTM | OURS | CNN-LSTM | CNN-Bi-LSTM | OURS |
| 0 | 0.0202 | 0.0179 | 0.0250 | 0.0070 | 0.0125 | 0.0201 |
| 30 | 0.0063 | 0.0028 | 0.0021 | 0.0042 | 0.0023 | 0.0010 |
| 60 | 0.0060 | 0.0028 | 0.0017 | 0.0040 | 0.0023 | 0.0008 |
| 90 | 0.0058 | 0.0028 | 0.0016 | 0.0039 | 0.0022 | 0.0008 |
| 120 | 0.0058 | 0.0027 | 0.0015 | 0.0038 | 0.0022 | 0.0008 |
| 150 | 0.0057 | 0.0027 | 0.0015 | 0.0038 | 0.0022 | 0.0008 |
| 180 | 0.0057 | 0.0026 | 0.0014 | 0.0037 | 0.0022 | 0.0008 |

**The data in Figure 11**

| Epochs | Training precision | | | Training precision | | |
| --- | --- | --- | --- | --- | --- | --- |
|  | CNN-LSTM | CNN-Bi-LSTM | OURS | CNN-LSTM | CNN-Bi-LSTM | OURS |
| 0 | 0.000 | 0.000 | 0.000 | 0.000 | 0.000 | 0.000 |
| 30 | 0.872 | 0.921 | 0.979 | 0.850 | 0.876 | 0.934 |
| 60 | 0.937 | 0.968 | 0.982 | 0.903 | 0.924 | 0.954 |
| 90 | 0.937 | 0.968 | 0.982 | 0.905 | 0.925 | 0.954 |
| 120 | 0.937 | 0.968 | 0.982 | 0.905 | 0.925 | 0.954 |
| 150 | 0.937 | 0.968 | 0.982 | 0.905 | 0.925 | 0.954 |
| 180 | 0.937 | 0.968 | 0.982 | 0.905 | 0.925 | 0.954 |

**The data in Figure 12**

| Time (min) | Time interval (5min) | | | | Time interval (10min) | | | |
| --- | --- | --- | --- | --- | --- | --- | --- | --- |
|  | Actual value | CNN-LSTM | CNN-Bi-LSTM | OURS | Actual value | CNN-LSTM | CNN-Bi-LSTM | OURS |
| 0 | 73 | 75 | 81 | 75 | 21 | 5 | 36 | 25 |
| 40 | 24 | 28 | 22 | 75 | 25 | 27 | 42 | 28 |
| 80 | 14 | 17 | 20 | 16 | 429 | 498 | 376 | 432 |
| 120 | 260 | 315 | 300 | 265 | 458 | 448 | 465 | 453 |
| 160 | 168 | 126 | 159 | 172 | 250 | 276 | 274 | 250 |
| 200 | 152 | 141 | 138 | 150 | 315 | 321 | 326 | 318 |
| 240 | 201 | 184 | 200 | 200 | 391 | 414 | 408 | 396 |
| 280 | 77 | 86 | 84 | 79 | 176 | 180 | 178 | 176 |

**The data in Figure 13**

| Model | MAPE | RMSE |
| --- | --- | --- |
| KNN | 0.277 | 27.83 |
| BPNN | 0.273 | 27.74 |
| VAR | 0.262 | 26.66 |
| SVR | 0.256 | 26.32 |
| CNN-Bi-LSTM | 0.242 | 24.85 |
| OURS | 0.233 | 23.87 |

**The data in Figure 14(a)**

| Time interval (min) | KNN | BPNN | VAR | SVR | CNN-Bi-LSTM | OURS |
| --- | --- | --- | --- | --- | --- | --- |
| 5 | 0.916 | 0.925 | 0.941 | 0.957 | 0.969 | 0.982 |
| 10 | 0.914 | 0.926 | 0.940 | 0.957 | 0.968 | 0.980 |
| 15 | 0.912 | 0.924 | 0.939 | 0.953 | 0.967 | 0.975 |
| 20 | 0.911 | 0.924 | 0.938 | 0.952 | 0.966 | 0.974 |

**The data in Figure 14(b)**

| Time interval (min) | KNN | BPNN | VAR | SVR | CNN-Bi-LSTM | OURS |
| --- | --- | --- | --- | --- | --- | --- |
| 5 | 0.918 | 0.931 | 0.946 | 0.962 | 0.974 | 0.988 |
| 10 | 0.915 | 0.931 | 0.943 | 0.960 | 0.973 | 0.984 |
| 15 | 0.916 | 0.928 | 0.941 | 0.957 | 0.971 | 0.982 |
| 20 | 0.911 | 0.927 | 0.940 | 0.955 | 0.968 | 0.979 |
